# Effects of metformin on transcriptomic and metabolomic profiles in breast cancer survivors enrolled in the randomized placebo-controlled MetBreCS trial

**DOI:** 10.1038/s41598-025-01705-9

**Published:** 2025-05-15

**Authors:** Pouda Panahandeh Strømland, Bjørn-Erik Bertelsen, Kristin Viste, Anastasia Chrysovalantou Chatziioannou, Federica Bellerba, Nivonirina Robinot, Amarine Trolat, Marianne Hauglid Flågeng, Augustin Scalbert, Pekka Keski-Rahkonen, Dorothy D. Sears, Bernardo Bonanni, Sara Gandini, Harriet Johansson, Gunnar Mellgren

**Affiliations:** 1 Hormone Laboratory, Department of Medical Biochemistry and Pharmacology, Haukeland University Hospital, Bergen, Norway; 2 Department of Clinical Science, University of Bergen, Bergen, Norway; 3 International Agency for Research on Cancer, Nutrition and Metabolism Branch, Lyon, France; 4 Department of Experimental Oncology, IEO, European Institute of Oncology IRCCS, Milan, Italy; 5 College of Health Solutions, Arizona State University, Phoenix, AZ USA; 6 Moores Cancer Center, University of California San Diego, La Jolla, San Diego, CA USA; 7 Department of Medicine, University of California San Diego, La Jolla, San Diego, CA USA; 8 Division of Cancer Prevention and Genetics, IEO, European Institute of Oncology IRCCS, Milan, Italy

**Keywords:** Metformin, Breast cancer recurrence, Sex steroid hormones, 17β-estradiol, Estrone, Metabolomics, Breast cancer, Transcriptomics, Steroid hormones, Cancer metabolism, Metabolomics

## Abstract

Metformin reduces the incidence of breast cancer in patients with obesity and type 2 diabetes. However, our knowledge of the effects of metformin on breast cancer recurrence is limited. Within the randomized double-blind placebo-controlled phase II trial MetBreCS, we examined changes in breast tissue from breast cancer survivors with BMI > 25 kg/m2 after treatment with metformin. To identify metformin-regulated signaling pathways, we integrated the transcriptomic, metabolomic and steroid hormone profiles using bivariate and functional analyses. We identified *MS4A1*, *HBA2*, *MT-RNR1*, *MT-RNR2*, *EGFL6* and *FDCSP* expression to be differentially expressed in breast tissues from metformin-treated postmenopausal women. The integration of transcriptomic and metabolomic profiles revealed down-regulation of immune response genes associated with reduced levels of arginine and citrulline in the metformin-treated group. The integration of transcriptomic and steroid hormone profiles showed an enrichment of steroid hormone biosynthesis and metabolism pathways with highly negatively correlated *CYP11A1* and *CYP1B1* expression in breast tissue from postmenopausal metformin-treated women. Our results indicate that postmenopausal breast cancer survivors treated with metformin have specific changes in breast tissue gene expression that may prevent the development of new tumors.

**Trial registration**: MetBreCs trial is registered at European Union Clinical Trials Register (EudraCT Protocol # 2015-001001-14) on 07/10/2015.

**Supplementary Information:**

The online version contains supplementary material available at 10.1038/s41598-025-01705-9.

## Introduction

Obesity and type 2 diabetes (T2D) are associated with an increased risk of postmenopausal breast cancer^1,2^ as well as several other types of cancer^3–5^. Insulin mediates mitogenic effects^6^, especially in cells expressing high levels of insulin receptor such as breast cancer cells^7,8^. In addition, increased levels of insulin may indirectly deregulate tumorigenic signaling in epithelial cells by changing other modulators such as inflammatory cytokines, adipokines and sex hormones^9,10^.

Excess serum 17β-estradiol (E2) and estrone (E1) in postmenopausal women are associated with increased breast cancer risk^11^. Estrogens stimulate carcinogenesis through several mechanisms including destructive oxidative metabolites and specifically in breast tissue through genomic and non-genomic estrogen receptor signaling^12^. Insulin and estrogen signaling may synergically stimulate cell proliferation through activation of RAS/MAPK and PI3K/AKT pathways in different types of cancer cells^13,14^. The involvement of insulin signaling in tumorigenesis has raised the possibilities for adopting therapeutic strategies to target insulin signaling in breast cancer patients^15,16^. Metformin is a commonly used anti-diabetic drug on the European market for at least 50 years. Metformin is generally well-tolerated and has minimal side effects. Importantly, the drug has been shown to decrease the risk of development of several tumor types in T2D patients^17,18^. Treatment with metformin, in contrast to other anti-diabetic drugs such as insulin and sulfonylurea, lowers the risk of cancer-associated mortality^17,19,20^. Metformin activates AMPK, which stimulates glucose uptake and glycogen synthesis, and suppresses hepatic gluconeogenesis, thereby improving whole-body insulin sensitivity in T2D patients^21^. The antitumor activity of metformin has also been attributed to these effects. In non-diabetic early-stage breast cancer patients, administration of metformin significantly improved insulin sensitivity and reduced serum insulin levels^22^. Moreover, metformin has been shown to reduce body weight, serum cholesterol and leptin in breast cancer patients^22,23^.

Despite our knowledge of the action of metformin in breast cancer, the knowledge about the potential preventive effect of metformin on cancer recurrence is limited. Within the MetBreCS trial^24,25^, we aimed to study the transcriptome of breast tissue applying RNA sequencing for high-quality expression profiling of 36 pre- and postmenopausal breast cancer survivors assigned to one year treatment with metformin or placebo.

In this study (MetBreCS trial^26^), we hypothesized that metformin decreases proliferation of healthy mammary epithelial cells in obese breast cancer survivors^27^. We included 36 pre- and postmenopausal breast cancer survivors assigned to one year treatment with metformin or placebo. Since all the premenopausal participants were randomized to the metformin treatment arm, making the potential effect of metformin in premenopausal women implausible, the ultimate transcriptomic effects of metformin in breast tissue were examined at baseline and post-treatment in 26 samples from postmenopausal participants. Integration of gene expression profiles with systemic levels of steroid hormones and other metabolites enabled us to identify signaling pathways in breast tissue that are altered by metformin treatment.

## Results

### Clinical subject characteristics

**Fig. 1:**
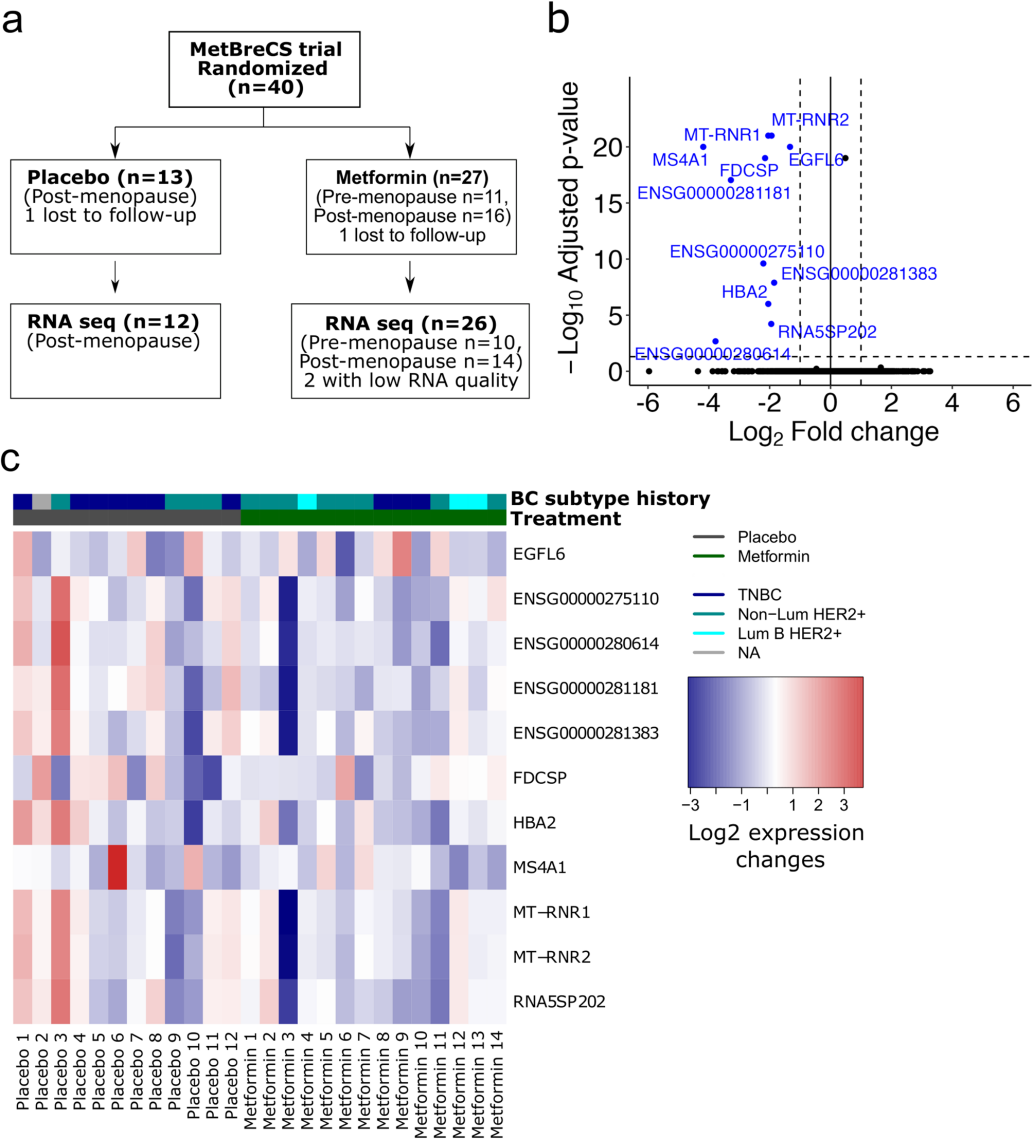
Metformin reduces the expression of tumorigenic genes *MS4A1*, *MT-RNR1/2* and *HBA2* in breast tissue. (**a**) Flow diagram of the pre- and postmenopausal women who participated in the MetBreCS trial. (**b**) Volcano plot showing deregulated genes comparing the transcriptomic profile of postmenopausal women treated with metformin (*n* = 14) vs. those treated with placebo (*n* = 12) using a time course likelihood ratio test analysis. The significant (adjusted *p* < 0.01) down-regulated genes (blue) are represented as log2 fold changes < 1.0 when comparing metformin vs. placebo. (**c**) Heatmap presenting the log2 gene expression changes (post-treatment vs. baseline) of the differentially expressed genes in panel B. The values are centered on the median of each gene expression change.

A flow diagram of the participants is presented in Fig. 1a and the characteristics of participants in the MetBreCS trial are shown in Supplementary Table 1. Of note, all the premenopausal participants were randomized to the metformin arm (Supplementary Table 1). We observed a difference in the baseline BMI between the placebo and metformin groups (Wilcoxon test, *p* = 0.019, Supplementary Table 1). However, at the end of the study, the treatment groups did not show any significant change in BMI. Therefore, the variations in the baseline BMI and menopause status were used as adjustments in multivariable analyses of metabolomics and steroid hormones data. Due to drop-out (*n* = 1), biopsy refusal at final visit (*n* = 1) and low RNA RIN (*n* = 2), four participants were excluded from the analyses. The primary endpoint of one-year metformin treatment in this study was changes in Ki-67 labeling index (LI) in contralateral unaffected breast biopsies. Given that we enrolled only 40 participants the study was under-powered for this biomarker, and the focus was therefore on secondary endpoints including, circulating metabolic biomarkers, steroid hormones, metabolomics, as well as tissue gene expression profiles. We have reported that analyses of cytokines and adipokines from the pooled Reach for Health (RFH) and MetBreCS trials, which included a total of 352 overweight/obese breast cancer survivors, demonstrated significant changes in leptin, sex hormone-binding globulin (SHGB) and C-reactive protein (CRP) levels following metformin and lifestyle interventions^24^. Since the postemenopausal participants in this study were evenly randomized to each treatment arm (Supplementary Table 1), we investigated the biomarker changes between the treatment groups exclusively in postmenopausal women participating in MetBreCS. We utilized multivariable linear regression models, adjusting for baseline biomarker values and baseline BMI to account for confounding factors. We observed a decreasing trend in leptin and CRP levels, an increasing trend for SHGB (Supplementary Table 2), and a significant positive correlation between *MKI67* expression and TNFα and resistin (Supplementary Fig. 1d).

### Metformin reduces the expression of *MS4A1*, *MT-RNR1/2* and *HBA2* genes in breast tissue of postmenopausal women

To examine how metformin affected the breast tissue gene expression, we obtained paired baseline and post-treatment RNA-sequencing profiles from the 36 pre- and postmenopausal breast cancer survivors. The principal component analysis of the gene expression profiles in breast tissue did not show a distinctive segregation between the placebo and metformin treatment groups (Supplementary Fig. 1a). This lack of segregation could be attributed to minimal changes in transcriptome profiles within each group. Given that the expression profiles in breast tissue derived from the pre- and postmenopausal women differ^28^, and all the premenopausal participants in this study were randomized to the metformin arm (Supplementary Table 1), further analyses were exclusively based on samples from postmenopausal women (Fig. 1b-c). Consequently, to investigate the differentially expressed genes after one-year of treatment with metformin compared to placebo, we conducted a supervised time course differential expression analysis of the global breast tissue transcriptomes. The results of the analysis using adjusted *p* < 0.01 and absolute log2 gene expression changes > 1.0 as cut-off are shown as volcano plot and heatmap (Fig. 1b-c, Supplementary Fig. 1b-c). We identified a set of differentially expressed genes including the *membrane-spanning 4-domains subfamily A member 1* (*MS4A1*), *mitochondrially encoded 12 S rRNA* (*MT-RNR1*), *mitochondrially encoded 16 S rRNA* (*MT-RNR2*) and *hemoglobin subunit alpha 2* (*HBA2*) that were highly down-regulated in the metformin-treated arm (Supplementary Fig. 1b-c and Fig. 1b-c). We also identified two genes, epidermal *growth factor-like domain-containing protein 6* (*EGFL6*) and *follicular dendritic cell secreted protein* (*FDCSP*), which were highly down-regulated after treatment with metformin compared to placebo in postmenopausal breast cancer survivors (Fig. 1b-c).

### Metformin-induced decrease in plasma arginine and citrulline are correlated with reduced immunity in breast tissue of postmenopausal women

**Fig. 2:**
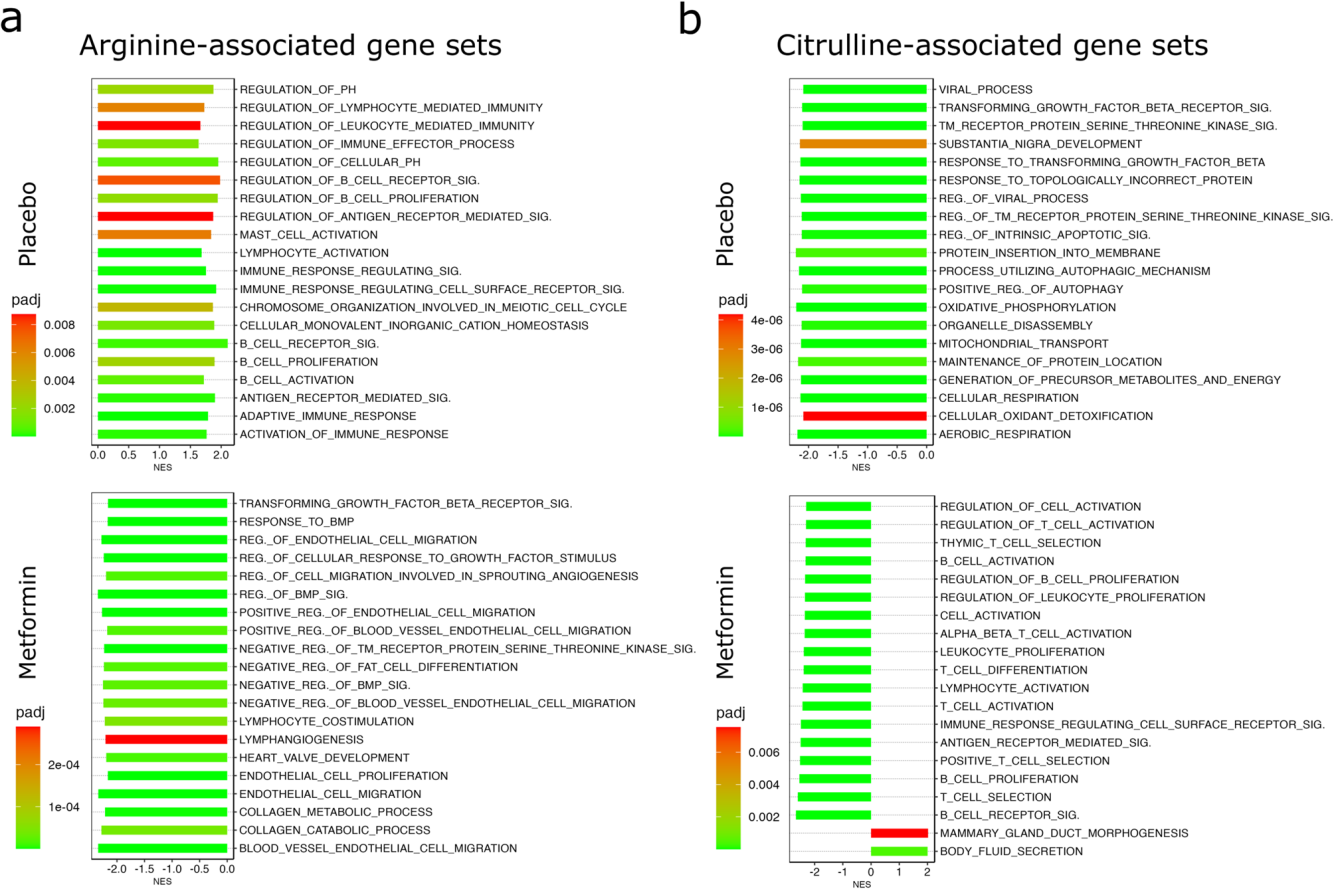
Metformin-induced decreases in plasma arginine and citrulline are associated with the down-regulation of immune response-related genes in postmenopausal women. (**a**) Functional analysis on the Spearman´s rank correlation between breast tissue gene expression changes (post-treatment vs., baseline) and plasma level changes (post-treatment vs. baseline) of the amino acid arginine revealed down-regulation of fat cell differentiation and endothelial proliferation and migration in the postmenopausal women treated with metformin compared to placebo. (**b**) Functional analysis on the Spearman´s rank correlation between breast tissue gene expression changes and plasma level changes of the amino acid citrulline revealed down-regulation of immune cell activation, proliferation and differentiation, and increased mammary gland morphogenesis in the postmenopausal women treated with metformin compared to those treated with placebo. The bar plots in panels A and B show the top 20 highly enriched pathways in placebo-treated and metformin-treated postmenopausal women, respectively. The bars represent the NESs. The significantly enriched pathways are shown as adjusted p-value < 0.01.

To examine the metabolite profile changes between the treatment groups, we applied multivariable linear regression models, adjusted for the metabolite baseline values, baseline BMI and menopause status as confounders (Supplementary Table 3). We observed an increased trend (β-regression coefficient > 0 and *p* < 0.05) in plasma hydroxybutyrylcarnitine and malonylcarnitine (C4-OH, C3-DC), asparagine, glycine, lysophosphatydilcholines lysoPCaC18:1 and lysoPCaC20:4 and a decreased trend (β-regression coefficient < 0 and *p* < 0.05) in plasma acylcarnitines C12 and C14:2, arginine, citrulline and several phosphatidylcholine species following metformin treatment (Supplementary Table 3). In line with a recently published pooled targeted metabolomics analysis of the RFH + MetBreCS trial^25^, the amino acids arginine and citrulline and the phosphatidylcholines PCaeC36:5 and PCaeC38:6 were significantly lower in the plasma of postmenopausal participants treated with metformin (Supplementary Tables 3 and Supplementary Fig. 2a). In the untargeted metabolomics data, we observed significant (*p* < 0.05) changes in features annotated as caffeine and 4-methyl-2-oxovalerate (Supplementary Tables 3 and Supplementary Fig. 2b) that were significantly altered in the pooled study^25^. We performed correlation analyses on these significantly altered metabolites in plasma and the transcriptomics of breast tissue for each postmenopausal treatment group, separately. Changes of arginine were highly correlated (coefficient > 0.8) with the expression of several breast tissue genes involved in immune cell activation and cell proliferation (Supplementary Fig. 2c-d). Arginine is synthesized from citrulline and acts as a direct activator of mTOR that strongly activates proliferation and metastasis of the cancer cells^29^. Arginine metabolism is also important in regulating immune responses of the tumor-infiltrating lymphocytes^30^. Therefore, using the correlation coefficient parameters from the correlation analyses, we further implemented the functional GSEA^31^ for arginine and citrulline for postmenopausal women (Fig. 2a-b). Compared to the placebo-treated group, gene sets representing fat cell differentiation and endothelial proliferation and migration were associated with reduced levels of plasma arginine in the postmenopausal metformin-treated women (Fig. 2a). Similarly, the functional analysis of genes correlated with plasma citrulline levels revealed a reduction in immune cell activation, proliferation and differentiation, alongside an increase in mammary gland morphogenesis in postmenopausal women treated with metformin compared to those given a placebo (Fig. 2b).

### Serum E1 and E2 are significantly correlated with specific plasma metabolites in metformin-treated postmenopausal women

**Fig. 3:**
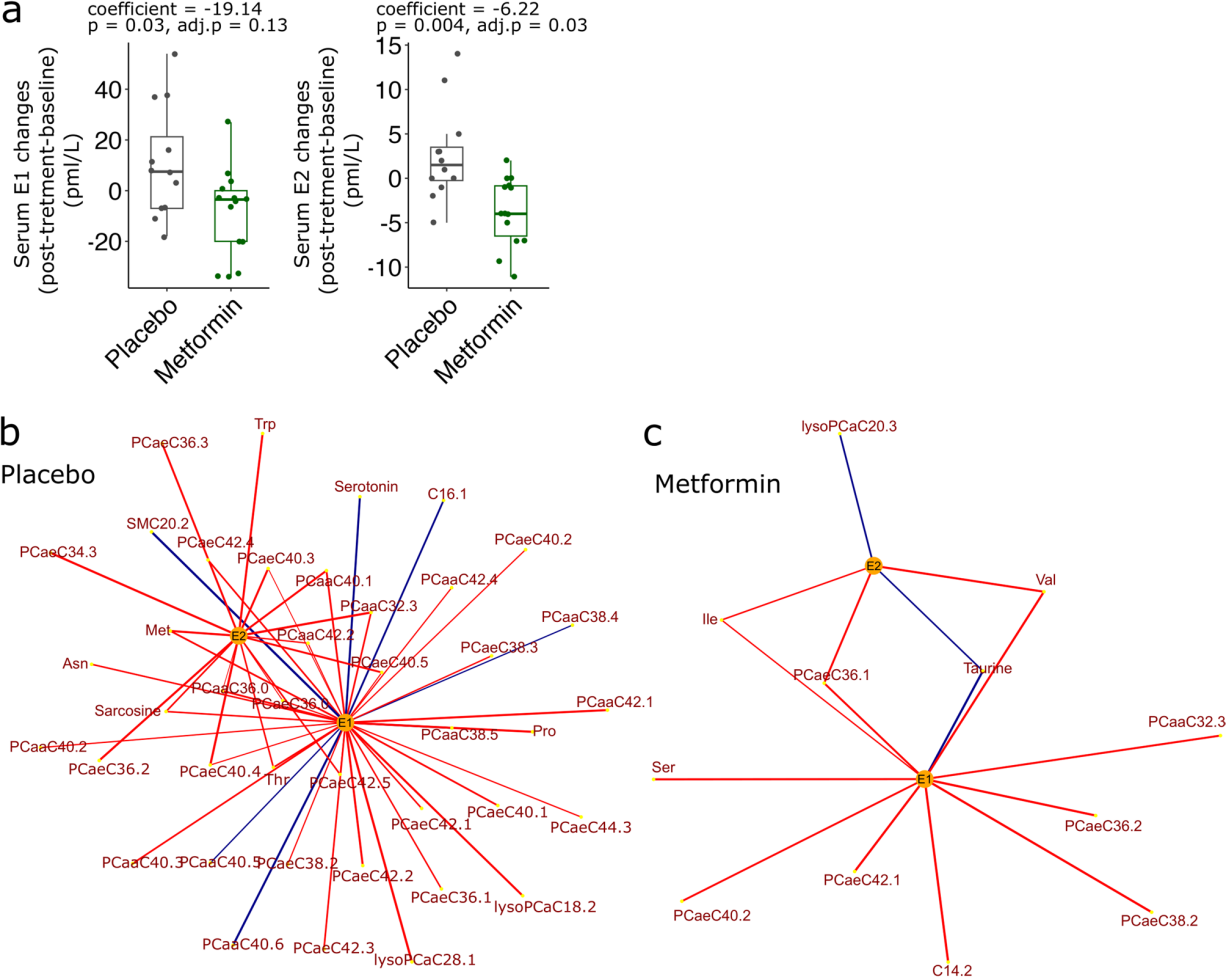
Changes in serum estrogens are associated with plasma metabolite changes in postmenopausal metformin-treated women. (**a**) The box plots represent changes (post-treatment vs. baseline) in serum E1 and E2 in the postmenopausal women treated with placebo or metformin in the MetBreCS trial. The negative β-regression coefficients derived from the multivariable linear model fit on these changes, adjusted for the baseline value of each steroid hormone and baseline BMI, indicate a decrease in serum E1 and E2 in the metformin arm. The p-value of the treatment covariate (Metformin vs. Placebo) from the multivariate linear model fit and the FDR-corrected p-value are indicated above the graphs. (**b**,**c**) Highly correlated plasma metabolite changes (post-treatment vs. baseline) with serum E1 and E2 changes in postmenopausal placebo- (panel B) and metformin-treated (panel C) groups. Positively and negatively correlated metabolites are represented by blue and red lines, respectively. The thickness of the lines indicates the correlation coefficient values (Spearman´s correlation coefficient > 0.5, *p* < 0.05).

Using newly developed ultra-sensitive LC-MS/MS assays^32,33^, we observed reduced levels of plasma E1 and E2 following metformin treatment in postmenopausal participants. These reductions were statististically significant (*p* < 0.05) with E2 showing a particularly notable decrease (adj. *p* = 0.03, Fig. 3a)^24^. Estrogen is a major effector in regulation of energy balance and metabolism^34^. Therefore, metformin-associated changes in serum E1 and E2 might affect the systemic metabolomic profiles. To investigate this, we correlated the changes of estrogens and metabolites in plasma from postmenopausal participants after treatment with metformin (Fig. 3b-c). Plasma taurine and lysoPCaC20:3 levels were positively correlated with the changes of both E1 and E2, whereas isoleucine was negatively correlated with the changes in serum levels of E1 and E2 (Fig. 3c). Moreover, sarcosine, methionine, asparagine, threonine and several phosphatidylcholines were negatively correlated with the changes of plasma E1 and E2 in the placebo arm (Fig. 3b). Among estrogen-correlated metabolites, we observed significantly increased asparagine (β-regression coefficient > 0, *p* < 0.05) and decreased levels of phosphatidylcholines PCaaC40:2, PCaaC40:3, PCaaC42:2, PCaaC42:4, PCaeC38:3, PCaeC40:3, PCaeC40:4, PCaeC42:4 (β-regression coefficient < 0, *p* < 0.05) compared to placebo-treated group (Supplementary Tables 3 and Fig. 3c).

We also examined potential associations between E1, E2 and other significantly modified metabolites and the differentially expressed genes that were identified. The changes in E1 and E2 were positively correlated (*p* < 0.05) with changes in the expression of *MT-RNR1/2*, *HBA2* and *RNA5SP202* in the postmenopausal metformin-treated women (Supplementary Fig. 3a). While changes in most of the plasma metabolites were negatively correlated to the corresponding gene transcripts in the placebo arm, the changes in expression of *HBA2* and *EGFL6* were negatively (*p* < 0.05) correlated with the changes of acylcarnitine C14.2 and arginine, respectively, in the metformin-treated women (Supplementary Fig. 3a). Similarly, we performed correlation analysis between all the quantified circulating levels of adipokines and cytokines, and the identified differentially expressed genes. The change in serum IGFBP-3 was positively correlated with the expression of *MT-RNR1*/2 in the postmenopausal metformin-treated group (Supplementary Fig. 3b).

### Expression of steroid metabolism genes ***CYP11A1*** and ***CYP1B1*** are negatively associated with serum E2 in metformin-treated postmenopausal women

**Fig. 4:**
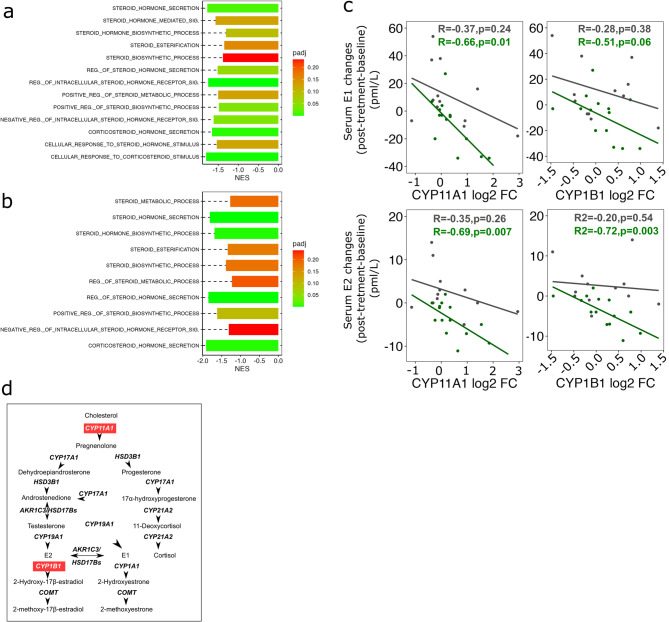
Serum estrogens are associated with the steroid metabolism pathway in breast tissue from metformin-treated postmenopausal women. (**a**,**b**) Functional analysis of the Spearman´s rank correlation between breast tissue gene expression changes (post-treatment vs. baseline) and serum level changes (post-treatment vs. baseline) of the steroid hormones E1 (a) and E2 (b) revealed enrichment of steroid hormone biosynthesis and metabolism pathways in the postmenopausal metformin-treated group. The bars represent NESs. The significant enriched pathways are shown as adjusted p-value (p-adj) < 0.25. (**c**) Significantly correlated *CYP11A1* and *CYP1B1* gene expression changes and serum level changes of E1 and E2. The linear regression lines are plotted for placebo- (gray) and metformin-treated (green) groups. (**d**) Summary of the steroid biosynthesis and metabolism pathway (adapted from KEGG^92,93^ hsa00140 with permission from Kanehisa Laboratories). CYP11A1 catalyzes the conversion of cholesterol to pregnenolone, the first and rate-limiting step in steroid biosynthesis. CYP1B1, a major E2 hydroxylase, catalyzes the metabolism of E2 to catechol estrogens (2-OHE2 and 4-OHE2) and highly reactive estrogen quinones (E2-2, 3-Q and E2-3, 4-Q)

To investigate potential effects of metformin on the expression of the genes regulating steroid hormone metabolism in postmenopausal women, we combined serum steroids with breast tissue transcriptomic data, using Spearman’s correlation analysis. Based on the correlation´s coefficients between the gene expression and the serum levels of E1 and E2, we generated ranked lists of genes and performed functional gene set enrichment analysis (GSEA). Interestingly, steroid hormone biosynthesis and metabolism pathways in the breast tissue from postmenopausal metformin-treated women were highly enriched (Fig. 4A-B). Further analysis of the GSEA data revealed that the expression of *CYP11A1*, encoding the first and rate-limiting enzyme in the steroid biosynthesis pathway, was negatively correlated with changes in serum E1 and E2 levels (Fig. 4C-D and Supplementary Fig. 4A). A similar negative correlation was observed between E2 levels and the expression of the *CYP1B1*, which encodes a major E2 metabolizing enzyme, catalyzing the conversion of E2 to catechol estrogens (2-OHE2 and 4-OHE2) and highly reactive estrogen quinones (E2-2, 3-Q and E2-3, 4-Q) (Fig. 4C-D and Supplementary Fig. 4A). Comparing the gene expression profiles (metformin vs. placebo), we observed an increased trend in the expression of *CYP11A1* and *CYP1B1*, in the metformin-treated postmenopausal subgroup (data not shown). In conclusion, we found a negative correlation between the expression of genes involved in steroid biosynthesis and metabolism pathways in breast tissue and serum levels of E1 and E2.

## Discussion

Metformin enhances cancer-related survival and reduces risk in diabetic patients with various types of cancer, particularly breast, colorectal, ovarian and endometrial cancers^35,36^. In breast cancer patients with T2D, use of metformin during neo-adjuvant chemotherapy has inhibitory effects on tumor progression^37^. Metformin also reduces the tumor growth in non-diabetic patients with obesity^23,38^. We can anticipate that the impact of an anti-diabetic drug, which alters immune response and various metabolites in MetBreCS^24,25^ may vary across breast cancer subtypes with distinct metabolic and immune profiles^39,40^. Several clinical studies have demonstrated that the positive effect of metformin are primarily observed in estrogen and progesterone receptor-positive, and HER2- negative breast cancer subtypes^41–43^. In the present study, most of the participants were non-diabetic, overweight or obese breast cancer survivors, and the biopsies were taken from breast tissues rather than the tumor tissues. Since there were no significant differences in the tumor subtypes between the treatment arms, our study could not differentiate the preventive effect of metformin specific to each breast cancer subtype.

As far as we know, our study is the first to investigate whether metformin may have a preventive effect on the initiation of new tumor lesions in human breast tissue by RNA sequencing analysis. By comparing metformin vs. placebo-treated breast tissue transcriptomic profiles, we identified several down-regulated genes including protein-encoding genes *MS4A1*, *HBA2*, and two mitochondrial genes *MT-RNR1/2*. Changes in *MS4A1* expression in breast tumors have been related to lower lipid metabolism and better survival^44^. *MS4A1* also encodes a B cell and a T cell subset surface marker (CD20) and is closely related to the immune-active tumor microenvironment and immune cell activation-related pathways in breast cancer^44^. Hemoglobin genes including hemoglobin subunit β (*HBB*) and *HBA2* are biomarkers of inflammatory breast cancer^45^. A transcriptomics study on the blood cells of the healthy individuals showed that short-term administration of metformin decreased the expression of *HBA* and *HBA2*^46^, an effect that we also observed in our study on breast tissue of the breast cancer survivors. The transcripts encoded by *MT-RNR1/2* genes have been associated with hepatocellular carcinoma and triple negative breast cancer progression^47^. The two *MT-RNR1* and *MT-RNR2* not only encode ribosomal RNA 12 S and 16 S, respectively, but are also transcribed to small proteins that are crucial for metabolic homeostasis. *MT-RNR1* encodes a peptide called mitochondrial open reading frame of the 12 S rRNA-c (MOTS-C) that is known to regulate insulin sensitivity through AMPK signaling pathway^48^. *MT-RNR2* also encodes a conserved cyto-protective polypeptide called humanin that promote tumor progression and metastasis by its anti-apoptotic effects^47^.

Whether overweight and obesity increase the risk of breast cancer in premenopausal women is controversial^49^. In the present clinical trial, all the premenopausal breast cancer survivors were randomized to the metformin-arm, preventing us from studying the potential molecular effects of metformin in the premenopausal overweight/obese breast cancer survivors.

In the differential analysis where changes in expression exclusively from postmenopausal breast tissue samples were examined, we found two additional down-regulated oncogenes, *EGFL6* and *FDCSP*. *EGFL6* is highly expressed in various malignant tumors compared to normal tissues^50,51^. EGFL6 is associated with cancer cell proliferation, migration, invasion and angiogenesis^52^, and promotes epithelial to mesenchymal transition of the cancer stem cells^53^, a mechanism targeted by metformin in breast tumors^54,55^. FDCSP is highly expressed in epithelial ovarian, breast, endometrial and prostate cancer types^56^ and contributes in the cancer cell invasion and metastasis through AKT signaling and down-regulation of E-cadherin^56^. Despite the limited knowledge of how these genes might be involved in regulation of cancer initiation and tumorigenesis, these transcriptomics data suggests that metformin might affect expression of breast tissue genes that precludes development of new tumors.

One potential mechanism of metformin action at the cellular level is disrupting mitochondrial function, thereby resulting in alterations of citric acid cycle^57^, oxidative phosphorylation as well as ATP production^58^. Here, we explored the potential associations between metabolites and breast tissue gene expression patterns in metformin-treated breast cancer survivors^25^. Using targeted metabolomics on the plasma samples, we observed metformin-associated changes in acylcarnitines, amino acids, lysophosphatidylcholines and several phosphatidylcholines, some of which were also reported to be similarly changed following metformin administration in the previous pooled RFH + MetBreCS study^25^. Carnitine and acylcarnitines are essential compounds for the oxidative metabolism of fatty acids in the mitochondria. Abnormal acylcarnitine metabolism has been associated with insulin resistance, obesity and breast cancer^59,60^. In contrast to the pooled RFH + MetBreCS study^25^, we found a slight metformin-associated reduction in malonylcarnitine/hydroxybutyrylcarnitine, and an increase in acylcarnitines dodecanoylcarnitine and tetradecadienylcarnitine, which are all known to be increased in plasma and urine from pre-diabetic and T2D individuals^61,62^. We also found a decreased trend in the plasma levels of long-chain unsaturated phosphatidylcholine species, two of which (PC ae C36:5 and PC ae C38:6) were also significantly decreased with the metformin treatment in the pooled RFH + MetBreCS study^25^. PC ae C38:6 has been reported to be strongly lowered by metformin treatment in patients with T2D^63^. Metformin also reduces cellular levels of several lysophosphatidylcholines, such as lysoPC a C16:0, lysoPC a C18:0, lysoPC a C18:1 and lysoPC a C18:2^64,65^. However, in our study, two lysophosphatidylcholines (lysoPC a C18:1 and lysoPC a C20:4) were increased in the plasma of metformin-treated breast cancer survivors. It should be noted that plasma measurements do not necessarily correspond to breast cells microenvironment. Whether the metformin-associated changes in the plasma levels of phosphatidylcholines correlate with the actual concentrations within the breast tissue remains to be examined.

Arginine, which is synthesized from citrulline regulates metabolic processes such as synthesis of polyamines and nitric oxide^66^, the latter of which modulates different cancer-related events^67^. Plasma arginine level is also associated with enhanced innate and adaptive immune responses in the tumors^68,69^. Dietary supplementation with L-arginine alone or in an adjuvant setting in patients with breast cancer significantly enhances host defenses by natural killer cells and activated lymphocyte cell cytotoxicity^70,71^. On the other hand, arginine deprivation leads to decreased cell growth and proliferation, and cell death in many tumor types^29,72^. Compared to the placebo-treated group, the functional analysis revealed enrichment of reduced immune responses, mitochondrial oxidative phosphorylation and endothelial cell proliferation and migration pathways associated with decrease in plasma arginine and citrulline in metformin-treated group.

Elevated serum E2 is linked to increased risk of breast cancer particularly in obese postmenopausal women^11,73^. After menopause, estrogens are mainly generated by extragonadal organs through conversion of androstenedione and testosterone to E1 and E2, which is catalyzed by the aromatase, encoded by *CYP19A1*^74^. Deregulation of serum concentrations of E1 and E2, mainly through the higher activity of aromatase enzyme in the visceral adipose tissue combined with decreased liver production of SHBG, has been suggested as a mechanism leading to increased risk of breast cancer in postmenopausal women with obesity and T2D^75,76^. In line with previous studies, we show that administration of metformin to postmenopausal breast cancer survivors reduces the serum levels of E2^77–79^. The increased expression of LKB1 and activity of AMPK, cytoplasmic sequestering of CREB and consequently down-regulation of the aromatase expression in the adipose tissue might explain the reduced levels of estrogen in the metformin-treated postmenopausal women^80,81^. There are also evidence suggesting that metformin targets the *CYP19A1* promoter^82^. In our RNA seq data, we were not able to detect any changes in *CYP19A1* expression in breast tissue from metformin-treated women. We observed negative correlations between the *CYP19A1* expression and changes in serum androstenedione and testosterone in the placebo- and metformin-treated arms, respectively, but no correlations between *CYP19A1* and changes in serum E1 or E2 (Supplementary Fig. 4A). Nevertheless, the expression of *CYP11A1* and *CYP1B1* were negatively correlated with the changes in serum E1 and E2 only in the metformin-treated postmenopausal group. The negative correlations between *CYP11A1* and *CYP1B1* expression and E1 and E2 might suggest a feedback mechanism to increase the biosynthesis and further metabolism of the estrogens. Although we did not find any significant changes in *CYP11A1* and *CYP1B1* expression in the breast tissue of the metformin-treated group, it has been reported that metformin is able to directly reduce the protein levels of CYP11A1 in the ovary granulosa cells^83^ and CYP1B1 in breast cancer cells^84^.

The present study has several limitations; first, the transcriptomics analysis is performed on breast tissue, and we may not conclude on cell-specific metformin effects. Moreover, the analyses of sex hormones and metabolites are limited to the systemic levels and do not provide a tissue-specific perspective. However, the integration of the systemic E2 and the altered metabolites with the corresponding gene expression profiles in the breast tissue provides an overall view in the treatment effect of metformin. Furthermore, the functional analyses were performed based on correlation analyses with no adjustment for any confounding factors such as baseline values and BMI and cannot prove causality but only show associations. Lastly, the small sample size in MetBreCS trial limits the statistical power in our analysis.

## Materials & methods

### Clinical study design, randomization and data collection

The MetBreCS trial is a randomized double-blind placebo-controlled phase II trial and was registered at the European Union Clinical Trials Register (EudraCT Protocol #: 2015-001001-14) on 07/10/2015. The trial was conducted at the European Institute of Oncology (IEO), Milan, Italy, and included overweight and obese (BMI > 25 kg/m2) breast cancer survivors (aged 18–70) with a previous diagnosis of triple negative, or ER-negative PgR-negative HER2-positive, or Luminal B HER2 positive breast cancer subtypes, without evidence of residual disease. Participants were randomly allocated to metformin or placebo in a 2:1 ratio, for one year of treatment. Metformin and placebo were manufactured to obtain the same pharmaceutical form, taste, shape, and color and assembled with the same type of packaging to ensure double blinding of the study drug. All the clinical and laboratory investigators involved in the study and the participants were blinded to treatment assignment.

The participants in the active treatment arm started with one tablet of 850 mg of metformin for the first three days, followed by two tablets of 850 mg for one-year. The results are presented in accordance with the Consolidated Standards of Reporting Trials (CONSORT) guidelines^85^. Fasting blood specimens and contralateral breast biopsies were collected at study entry (baseline) and at the final one-year post-treatment visits. As previously reported^24^ the MetBreCS did not reach the desired sample size due to recruitment difficulties and recruitment was halted at the end of 2018. Notably, since all premenopausal participants were randomized to the metformin treatment arm, the effects of metformin in this study were exclusively examined in postmenopausal participants. The analyses aimed to efficiently explore RNA expression transcriptomics in breast tissue, modulation by metformin compared to placebo, and to correlate these findings with secondary outcomes in the available blood specimens from postmenopausal breast cancer survivors^24,25^. Recruitment to MetBreCS trial was conducted between January 2017 to November 2018 and the follow-up ended in November 2019.

### Ethics approval and consent to participate

The local institutional review board at the European Institute of Oncology (IEO), Milan, approved the MetBreCS trial, and participants signed informed consent. The research on MetBreCS trial was carried out in accordance with the international standards for good clinical and laboratory practice.

### Tissue RNA extraction and RNA sequencing

RNA from snap-frozen breast tissue biopsies were purified after lysing by tissuelyser (Qiagen) and using RNA Purification Plus Kit (Norgen biotek CORP, 47700) with additional on-column DNase-I treatment (Qiagen, 79254) at 27 °C. RNA purity and integrity (RIN) were quantified using RNA 6000 Nano kit (Agilent Technologies, 5067 − 1511) on the 4200 TapeStation (Agilent, Santa Clara, USA). Library preparation and 2 × 75 bp paired-end of 160 ng total RNA input was performed using Illumina Stranded Total RNA Prep Ligation kit and Illumina HiSeq4000 system (Illumina, Sand Diego, CA, USA). RNA sequencing data from HiSeq4000 were quality checked and aligned to GRCh38 (GCA_000001405.15) reference genome using HISAT2 2.0.5 and submitted to subread v.1.5.2 for feature counts calculation. A total of 36 paired biopsies samples were sequenced: 26 from the metformin group (10 premenopausal and 14 postmenopausal participants) and 12 from the placebo group). Four paired samples were excluded due to drop-out (*n* = 1), biopsy refusal at final visit (*n* = 1) and low RNA RIN (*n* = 2). The final transcriptomic analyses were conducted exclusively on data from 26 postmenopausal participants. These number of samples provided a sufficient and powerful dataset to explore the molecular changes in the participants’ breast tissue in two treatment arms. The raw read count was then submitted to DESeq2 (Version 1.24.0)^86^, normalized and filtered. To capture the greatest variation between the metformin and placebo treatments, we first performed principle component analysis (PCA). Subsequently, differentially expressed genes between the metformin and placebo treatments were identified using a time course likelihood ratio test (LRT) to detect genes that changed differentially after one year of treatment. The differential expressed genes were selected using a Benjamini-Hochberg (BH)-adjusted *p* < 0.01 and an absolute log2 fold change > 1.0.

### Metabolomics analyses

Details of the targeted and untargeted metabolomics data acquisition and processing have been provided in a recent publication^25^. In total, 145 and 703 metabolite features were obtained in targeted and untargeted metabolomics, respectively, for the samples in the MetBreCS trial. For both metabolomics data sets, the changes (post-treatment vs. baseline) in the levels of metabolites after log-transformation, imputation and scaling were compared using multivariable linear regression models on the paired samples with the available transcriptomics data between treatment groups (metformin (*n* = 24) vs. placebo (*n* = 12)). Models were fit on the scaled metabolite changes and adjusted for the scaled baseline value, baseline BMI and menopause status (Supplementary Table 1). All p-values were adjusted for false discovery rate (FDR) through BH procedure. We considered *p* < 0.05 as statistically significant for the primary analyses of MetBreCS data sets. Next, we confirmed the altered metabolites with the previously published pooled Reach For Health (RFH) + MetBreCS data sets before further analyses^25^. All statistical analyses were performed using R^87^.

### Steroid hormone and biomarker analyses

Serum samples were analyzed for steroid hormones including E1 and E2 using two previously reported ultra-sensitive LC-MS/MS assays^32,33^. The methods for analyzing serum adiponectin, leptin, resistin, complement factor D, CCL2, Serpin/PAI-1, IL-6, IL-10, TNF-α, IGF-1, IGFBP-3, SHBG, insulin, CRP and HOMA-IR have been detailed in a recent publication^24^.

### Transcriptomic, metabolomic and steroid hormone data bivariate and functional analyses

Spearman´s rank correlation test was primarily used to perform bivariate analysis between the steroid hormones E1 and E2 and metabolomics obtained from the multivariable linear regression models, and the transcriptomics data for the patient groups (postmenopausal placebo and metformin), separately. The normalized gene expression changes (log2 (post-treatment/baseline)) for each group were correlated with the corresponding plasma metabolite changes. Further, the correlation coefficients with significant *p* < 0.05 were used to generate interaction networks using igraph^88^ R package. The coefficient > 0.8 and > 0.5 were used in the interaction networks for gene expression vs. metabolites, and steroid hormones vs. metabolomics data, respectively. The obtained correlation coefficients and the corresponding p-value were further used to build a ranked list of genes to run functional analysis using gene set enrichment analysis (GSEA)^31^ for the enrichment of gene ontology:biological process (GO:BP) gene sets available at molecular signature database (MsigDB)^89^ using msigdb^90^ and fgsea^91^ packages. The gene sets with absolute normalized enrichment score (NES) > 1.5 and FDR < 0.25 were used as significant. The genes with standard deviation = zero gene expression were not considered in the correlation analysis.

### Statistical methods

The differences between the treatment groups (Supplementary Table 1) were tested with Wilcoxon rank-sum or Chi-square tests for the numerical and categorical of the clinical features, respectively. P-values < 0.05 were considered significant for these tests. Other statistical methods are indicated in each respective section. All the statistical analyses were performed using R^87^.

## Electronic supplementary material

Below is the link to the electronic supplementary material.

Supplementary Material 1

## Data Availability

The steroid hormones, metabolomics and other biomarker data underlying this article may be shared upon reasonable request to the PI of the MetBreCS study (Dr. Bernardo Bonanni), following approval by the Data and Safety Monitoring Board at IEO, Milan. The gene expression data are publicly available at the European Genome-phenome Archive (EGA) under the accession ID number EGAC50000000523.
