# Supplementary material for: Effects of metformin on transcriptomic and metabolomic profiles in breast cancer survivors enrolled in the randomized placebo-controlled MetBreCS trial
Source: Sci Rep. 2025 May 15;15:16897. doi: 10.1038/s41598-025-01705-9 (PMC12081705; doi:10.1038/s41598-025-01705-9)
Supplement: Supplementary file 1 — Supplementary Material 1 [file 41598_2025_1705_MOESM1_ESM.docx]

**Effects of Metformin on Transcriptomic and Metabolomic Profiles in Breast Cancer Survivors Enrolled in the Randomized Placebo-Controlled MetBreCS Trial**

Pouda Panahandeh Strømland^1,2^, Bjørn-Erik Bertelsen^1^, Kristin Viste^1^, Anastasia Chrysovalantou Chatziioannou^3^, Federica Bellerba^4^, Nivonirina Robinot^3^, Amarine Trolat^3^, Marianne Hauglid Flågeng^1^, Augustin Scalbert^3^, Pekka Keski-Rahkonen^3^, Dorothy D. Sears^5,6,7^, Bernardo Bonanni^8^, Sara Gandini^4^, Harriet Johansson^8^, Gunnar Mellgren^1,2,*^

1. Hormone Laboratory, Department of Medical Biochemistry and Pharmacology, Haukeland University Hospital, Bergen, Norway

2. Department of Clinical Science, University of Bergen, Bergen, Norway

3. International Agency for Research on Cancer, Nutrition and Metabolism Branch, Lyon, France

4. Department of Experimental Oncology, IEO, European Institute of Oncology IRCCS, Milan, Italy.

5. College of Health Solutions, Arizona State University, Phoenix, AZ, USA

6. Moores Cancer Center, University of California San Diego, La Jolla, CA, USA

7. Department of Medicine, University of California San Diego, La Jolla, CA, USA

8. Division of Cancer Prevention and Genetics, IEO, European Institute of Oncology IRCCS, Milan, Italy

* Corresponding author; email: gunnar.mellgren@uib.no

**Supplementary information**





**Supplementary Fig. 1** a. Principal component analysis of the normalized gene expression profiles of the pre- and postmenopausal participants in the MetBreCS trial. Transcriptomic profile changes (post-treatment vs. baseline) are shown in two representative principal components (PC1 and PC2). Each color represents the treatment group (placebo and pre- and postmenopausal metformin). b. Volcano plot showing differentially expressed genes comparing the transcriptomic profiles of pre- (n=10) and postmenopausal (n=14) metformin-treated group vs. postmenopausal placebo-treated group (n=12) using a time course likelihood ratio test. Significant (adjusted p <0.01) over-expressed genes (red) are represented as log2 fold changes >1.0 when comparing metformin to. Placebo, and down-regulated genes (blue) are represented as log2 gene expression changes <1.0. c. Heatmap presenting the log2 gene expression changes (post-treatment vs. baseline) of the differentially expressed genes in panel b. The values are centered on the median of each gene expression change. d. The heat map illustrates Spearman´s correlation between *MKI67* gene expression and circulating levels of adipokines and cytokines (p <0.05) in the postmenopausal women treated with metformin- vs. placebo. The circle size and color represent the correlation coefficient, as indicated by the color bar. Significantly correlated genes (p <0.05) are marked with squares.





**Supplementary Fig. 2** a-b. The box plots illustrate significantly modified metabolites when comparing postmenopausal metformin- vs. placebo-treated groups (p <0.05), which were also identified in the pooled RFH + MetBreCS study using targeted (a) and untargeted (b) metabolomics approaches. The results of the multivariable linear model fit test (Metformin vs Placebo) are presented in Table S2. c-d. Highly correlated breast tissue gene expression (Spearman´s correlation coefficient >0.8, p <0.05) with metformin-associated plasma metabolites shown in panels a-b. cand d show the highly correlated gene transcripts in the postmenopausal placebo and metformin-treated groups, respectively. Positively and negatively correlated genes are represented by blue and red lines, respectively. The thickness of the lines indicates the correlation coefficient values.





**Supplementary Fig. 3** a. The heat map illustrates Spearman´s correlation between differentially expressed breast tissue genes and the circulating levels of significantly altered metabolites (p <0.05), E1 and E2 in the postmenopausal women treated with metformin versus Placebo. The circle size and color represent the correlation coefficient, indicated by the color bar. Significantly correlated genes (p <0.05) are marked with squares. b. The heat map shows Spearman´s correlation between differentially expressed breast tissue genes and circulating levels of adipokines and cytokines (p <0.05) in the postmenopausal women treated with metformin vs. placebo. The circle size and color represent the correlation coefficient, as indicated by the color bar. Significantly correlated genes (p <0.05) are marked with squares. c. The heatmap illustrates Spearman’s correlation between the transcriptional expression of *MKI67* in breast tissue and circulating levels of adipokines and cytokines (p <0.05) in the postmenopausal women treated with metformin vs. placebo. The circle size and color represent the correlation coefficient as shown by the color bar. Significantly correlated genes (p <0.05) are marked with squares.





**Supplementary Fig. 4** A. The heat map illustrates Spearman’s rank correlation between the expression of genes involved in steroid biosynthesis and metabolism and serum levels of E1 and E2 in the postmenopausal women treated with placebo or metformin. The sizes and colors of the circles represent the correlation coefficients. Significantly correlated genes are marked with squares. The genes with standard deviation equal to zero were excluded from this analysis

**Supplementary Tables 1** Characteristics of the participants by treatment groups in the MetBreCS trial

|  | | | |
| --- | --- | --- | --- |
|  | **Placebo** | **Metformin** | **p-value*** |
|  | **(N=13, one lost follow-up)** | **(N=27)** |  |
| **Weight Loss intervention n (%)** |  |  |  |
| No | 7 (58.3%) | 13 (48.2%) | 1 |
| Yes | 5 (41.7%) | 11 (40.7%) |  |
| Missing | 0 (0%) | 3 (11.1%) |  |
|  |  |  |  |
| **Age, median [Q1, Q3]** | 62 [48.3, 64.3] | 52 [47.3, 55.0] | 0.159 |
|  |  |  |  |
| **Baseline BMI, median [Q1, Q3]** | 28.1 [26.8, 29.3] | 29.5 [26.7, 31.4] | 0.019 |
|  |  |  |  |
| **Menopausal status, n (%)** |  |  |  |
| Post-menopausal | 12 (100%) | 16 (59.3%) | 0.025 |
| Pre-menopausal | 0 (0%) | 11 (40.7%) |  |
|  |  |  |  |
| **Tumor molecular subtype, n (%)** |  |  |  |
| Triple negative | 7 (58.3%) | 10 (37.0%) | 0.154 |
| Non-luminal HER2+ | 4 (33.3%) | 11 (40.7%) |  |
| Luminal HER2+ | 0 (0%) | 5 (18.5%) |  |
| Missing | 1 (8.3%) | 2 (7.4%) |  |
|  |  |  |  |
| **Estrogen Receptor Status, n (%)** |  |  |  |
| Negative | 12 (100%) | 21 (77.8%) | 0.233 |
| Positive | 0 (0%) | 6 (22.2%) |  |
| Missing | 0 (0%) | 0 (0%) |  |
|  |  |  |  |
| *p-values derived from Wilcoxon rank-sum test for numerical variables and from Chi-square test for categorical variables. | | | |
| Differences between the treatment groups (Metformin vs. Placebo) were tested using Wilcoxon rank-sum tests for the numerical clinical features and Chi-square tests for categorical clinical features | | | |

**Supplementary Table 2** Serum biomarkers analyzed in the MetBreCS trial showing metformin treatment-associated changes over time compared to placebo in postmenopausal women

| **Biomarker (unit)** | **β-regression coefficient *** | **p-value **** |
| --- | --- | --- |
| CCL2 (pg/ml) | 26.17 | 0.49 |
| IL-10 (pg/ml) | 0.12 | 0.31 |
| IL-6 (pg/ml) | -0.23 | 0.63 |
| TNFα | 0.38 | 0.37 |
| Complement factor D (ng/ml) | -244.06 | 0.056 |
| IGF1 (ng/ml) | -2.02 | 0.75 |
| IGBP3 (ng/ml) | -172.24 | 0.17 |
| Adiponectin (ug/ml) | 0.09 | 0.09 |
| Leptin (ng/ml) | -10.6 | 0.27 |
| Serpin/PAI-1 (ng/ml) | 4.33 | 0.66 |
| Resistin (ng/ml) | -0.013 | 0.98 |
| SHBG (nmol/L) | 1.9 | 0.55 |
| Insulin (uU/ml) | -0.97 | 0.45 |
| CRP (mg/dL) | -0.085 | 0.28 |
|  |  |  |
| * A positive beta coefficient indicates a greater increase over time in the biomarker in the metformin arm compared to the placebo arm, whereas a negative coefficient indicates a greater decrease in the metabolite in the metformin arm. | | |
| ** The p-value of the treatment covariate (Metformin vs. Placebo) is derived from a multivariable linear regression model fit on the metabolite changes (post-treatment vs. baseline) adjusted for the basal value of each metabolite and baseline BMI. | | |

**Supplementary Table 3** Identified plasma metabolites with metformin treatment-associated changes over time that differ significantly from placebo (p <0.05)

|  | | | | |
| --- | --- | --- | --- | --- |
| **Metabolites/Features** | **Metabolite class/Annotated feature** | **β-regression coefficient *** | **p-value **** | **Adjusted p-value ***** |
| **Targeted approach** |  |  |  |  |
| C4-OH (C3-DC) | Hydroxybutyrylcarnitine | 0.012 | 0.02 | 0.33 |
| C12 | Dodecanoylcarnitine | -0.022 | 0.04 | 0.33 |
| C14:2 | Tetradecadienylcarnitine | -0.007 | 0.05 | 0.33 |
| **Arg** | **Amino acid** | **-9.447** | **0.03** | **0.33** |
| Asn | Amino acid | 4.779 | 0.01 | 0.33 |
| **Cit** | **Amino acid** | **-7.241** | **0.04** | **0.33** |
| Gly | Amino acid | 35.284 | 0.05 | 0.33 |
| lysoPCaC18:1 | Glycerophospholipids | 3.331 | 0.05 | 0.33 |
| lysoPCaC20:4 | Glycerophospholipids | 0.714 | 0.02 | 0.33 |
| PCaaC40:2 | Glycerophospholipids | -0.073 | 0.04 | 0.33 |
| PCaaC40:3 | Glycerophospholipids | -0.079 | 0.01 | 0.33 |
| PCaaC42:2 | Glycerophospholipids | -0.032 | 0.04 | 0.33 |
| PCaaC42:4 | Glycerophospholipids | -0.029 | 0.05 | 0.33 |
| PCaaC42:5 | Glycerophospholipids | -0.039 | 0.01 | 0.33 |
| PCaeC36:3 | Glycerophospholipids | -1.227 | 0.04 | 0.33 |
| **PCaeC36:5** | **Glycerophospholipids** | **-1.943** | **0.03** | **0.33** |
| PCaeC38:3 | Glycerophospholipids | -1.404 | 0.02 | 0.33 |
| **PCaeC38:6** | **Glycerophospholipids** | **-1.039** | **0.04** | **0.33** |
| PCaeC40:3 | Glycerophospholipids | -0.601 | 0.02 | 0.33 |
| PCaeC40:4 | Glycerophospholipids | -0.386 | 0.04 | 0.33 |
| PCaeC42:4 | Glycerophospholipids | -0.109 | 0.03 | 0.33 |
|  |  |  |  |  |
| **Untargeted approach ****** | |  |  |  |
| 246.1375@2.6396875 | not annotated | 134226.365 | 0.01 | 0.55 |
| 152.0577@1.6871359 | not annotated | 28979.189 | 0.04 | 0.85 |
| 519.3327@6.9529095 | LysoPC(18:2) | -95638.262 | 0.01 | 0.55 |
| 541.3152@6.9534364 | LysoPC(20:5) | -57464.578 | 0.04 | 0.85 |
| 186.0498@0.73312473 | not annotated | 55233.060 | 0.02 | 0.85 |
| **216.0625@3.214955** | **Caffeine** | **35499.548** | **0.04** | **0.85** |
| 279.1429@6.952684 | LysoPC(18:2) | -25291.183 | 0.01 | 0.55 |
| 543.3312@7.1253047 | LysoPC(20:4) | -25681.383 | 0.02 | 0.75 |
| 271.1536@6.9532104 | LysoPC(20:5) | -43318.851 | 0.01 | 0.65 |
| 136.0624@0.61867136 | 1-methylnicotinamide | 6579.261 | 0.01 | 0.55 |
| 177.9578@0.872926 | not annotated | -27679.691 | 0.02 | 0.75 |
| 287.1291@6.953939 | LysoPC(18:2) | -17923.941 | 0.02 | 0.79 |
| 178.9831@1.0052351 | not annotated | -10585.020 | 0.03 | 0.85 |
| 69.057@0.7127944 | not annotated | -10471.414 | 0.03 | 0.85 |
| 285.2226@7.241724 | not annotated | -6678.057 | 0.02 | 0.85 |
| **247.0282@3.1562908** | **4-methyl-2-oxovalerate** | **-7510.986** | **0.05** | **0.88** |
| 204.9804@0.815727 | not annotated | -8385.396 | 0.04 | 0.85 |
| 543.3319@7.1253843 | LysoPC(20:4) | -23031.422 | 0.04 | 0.85 |
| 473.2773@5.868209 | not annotated | 9590.035 | 0.04 | 0.85 |
| 247.1421@0.9074893 | not annotated | -2832.645 | 0.01 | 0.55 |
| 220.0237@1.744032 | not annotated | -11515.568 | 0.00 | 0.48 |
| 543.3316@7.1253843 | LysoPC(20:4) | -22669.818 | 0.03 | 0.85 |
| 343.2733@5.7722397 | not annotated | 4694.556 | 0.03 | 0.85 |
| 128.0479@0.7241638 | not annotated | 5138.513 | 0.04 | 0.85 |
| 187.1197@0.7376418 | not annotated | 2214.131 | 0.04 | 0.85 |
| 268.2196@7.238816 | Retinol | -2740.858 | 0.03 | 0.85 |
| 291.1413@6.942807 | not annotated | -4390.615 | 0.03 | 0.85 |
| 152.0586@1.6871792 | not annotated | 35025.480 | 0.02 | 0.75 |
| 224.103@5.027979 | not annotated | 2045.673 | 0.05 | 0.88 |
| 566.3189@6.9446955 | not annotated | -2852.299 | 0.03 | 0.85 |
| 57.0571@0.7027857 | not annotated | -2387.405 | 0.01 | 0.65 |
| 350.1191@0.73251426 | not annotated | 4173.648 | 0.00 | 0.48 |
| 114.0422@0.88638544 | 5,6-Dihydrouracil | 2775.583 | 0.01 | 0.55 |
| **366.113@0.727018** | **Unknown** | **3287.988** | **0.002** | **0.48** |
| 287.9146@0.5565221 | not annotated | 2288.350 | 0.02 | 0.75 |
| 181.0952@0.7358742 | not annotated | 3189.467 | 0.01 | 0.65 |
| 75.0322@0.8909828 | Glycine | -2428.762 | 0.03 | 0.85 |
| 139.061@0.8538033 | Valine | -8544.883 | 0.04 | 0.85 |
|  |  |  |  |  |
| * A positive beta coefficient indicates a greater increase over time in the metabolite in the metformin arm compared to the placebo arm, whereas a negative coefficient indicates a greater decrease in the metabolite in the metformin arm. | | | | |
| ** The p-value of the treatment covariate (Metformin vs. Placebo) is derived from a multivariable linear regression model fit on the metabolite changes (post-treatment vs. baseline) adjusted for the basal value of each metabolite, baseline BMI and menopause status | | | | |
| *** FDR-corrected p-value | |  |  |  |
| **** The features are described in the Mass@retention time (min) format. | | | | |
| The metformin-associated significant changes in metabolites found in the pooled RFH + MetBreCS study are in bold | | | | |
